# Supplementary material for: Hypoxia induces senescence of bone marrow mesenchymal stem cells via altered gut microbiota
Source: Nat Commun. 2018 May 22;9:2020. doi: 10.1038/s41467-018-04453-9 (PMC5964076; doi:10.1038/s41467-018-04453-9)
Supplement: Supplementary file 1 — Supplementary Information [file 41467_2018_4453_MOESM1_ESM.pdf]

Hypoxia induces senescence of bone marrow Mesenchymal stem cells via altered gut microbiota

Xing et al

Supplementary Information

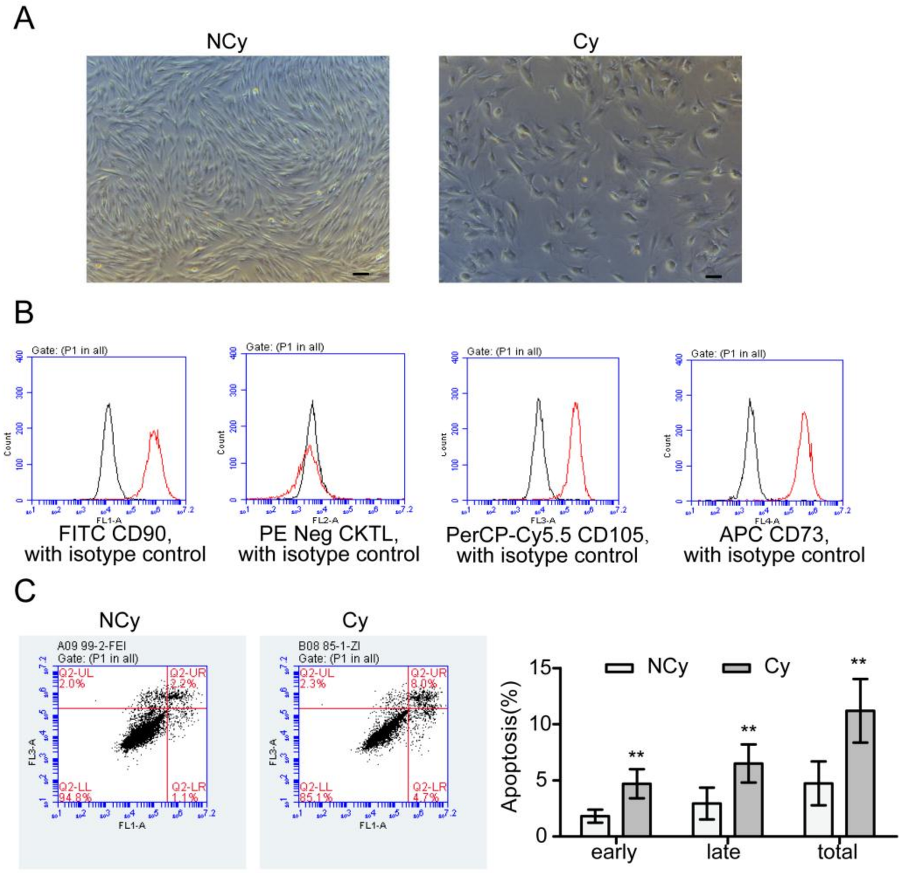

Supplementary Figure 1. Morphology and Phenotype of BMSCs. (A) Analysis of BMSC morphology in the NCy and Cy groups using an inverted microscope. Scale bar, 100μm. **(B)** Flow cytometry to examine the phenotype of BMSCs, all of which were positive for CD90, CD105, and CD73 and negative for CD34, CD45, CD11b, CD19, and HLA-DR. **(C)** BMSC apoptosis was quantified by flow cytometric analysis after staining with Annexin V and propidium iodide (PI). The Annexin V+/PI- cells were undergoing early apoptotic processes, and the Annexin V+/PI+ cells were undergoing late apoptotic processes. Data are the mean ± SD from 3 independent experiments, **P<0.01.

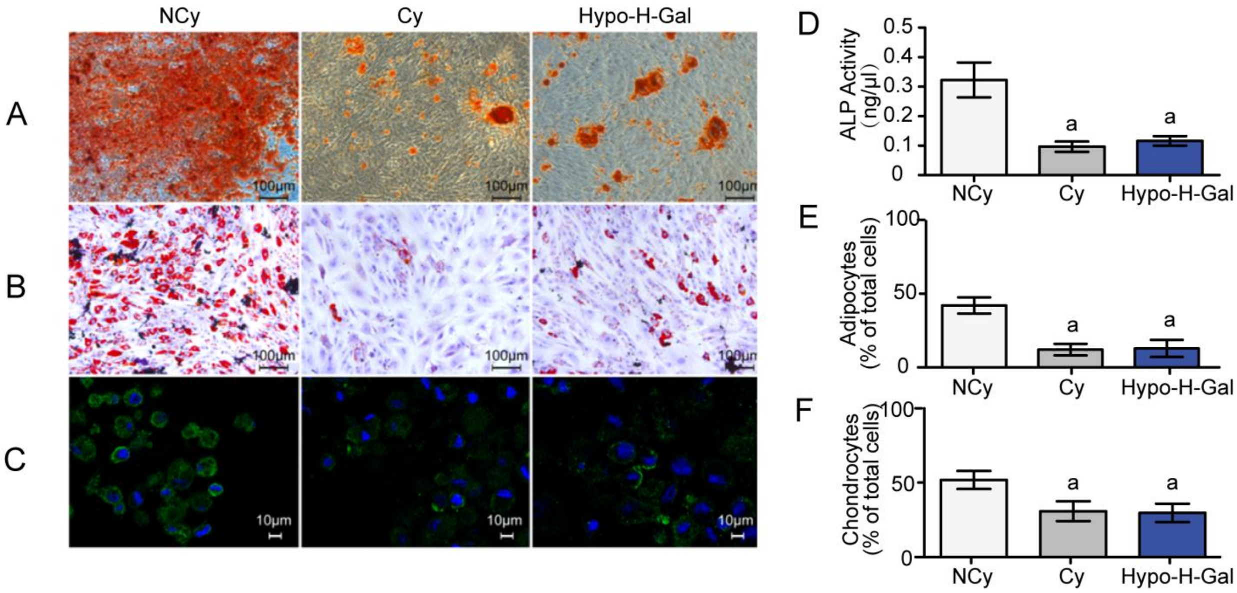

Supplementary Figure 2. BMSCs from patients with CCHD had impaired multilineage differentiation potential. (A, D) Osteogenic differentiation assay of BMSCs from the NCy, Cy, and Hypo-H-Gal groups (n=5 per group). Alkaline phosphatase (ALP) activity assay **(D)** and Alizarin red staining **(A)** were carried out to estimate osteogenic differentiation potential among the three groups. **(B, E)** Adipogenic differentiation assay of BMSCs from the NCy, Cy, and Hypo-H-Gal groups. Adipocytes among the three groups were stained with Oil Red O **(B)**, and the adipogenic differentiation ratio was calculated **(E)**. **(C, F)** Chondrogenic differentiation assay of BMSCs from the NCy, Cy, and Hypo-H-Gal groups. Chondrocytes in the three groups were detected by immunofluorescent staining for type II collagen **(C)**, and the chondrogenic differentiation ratio was calculated **(F)**. Data are the mean ± SD from 3 independent experiments, and statistical significance was analysed using the Student's t test (a $P < 0.05$ compared with NCy).

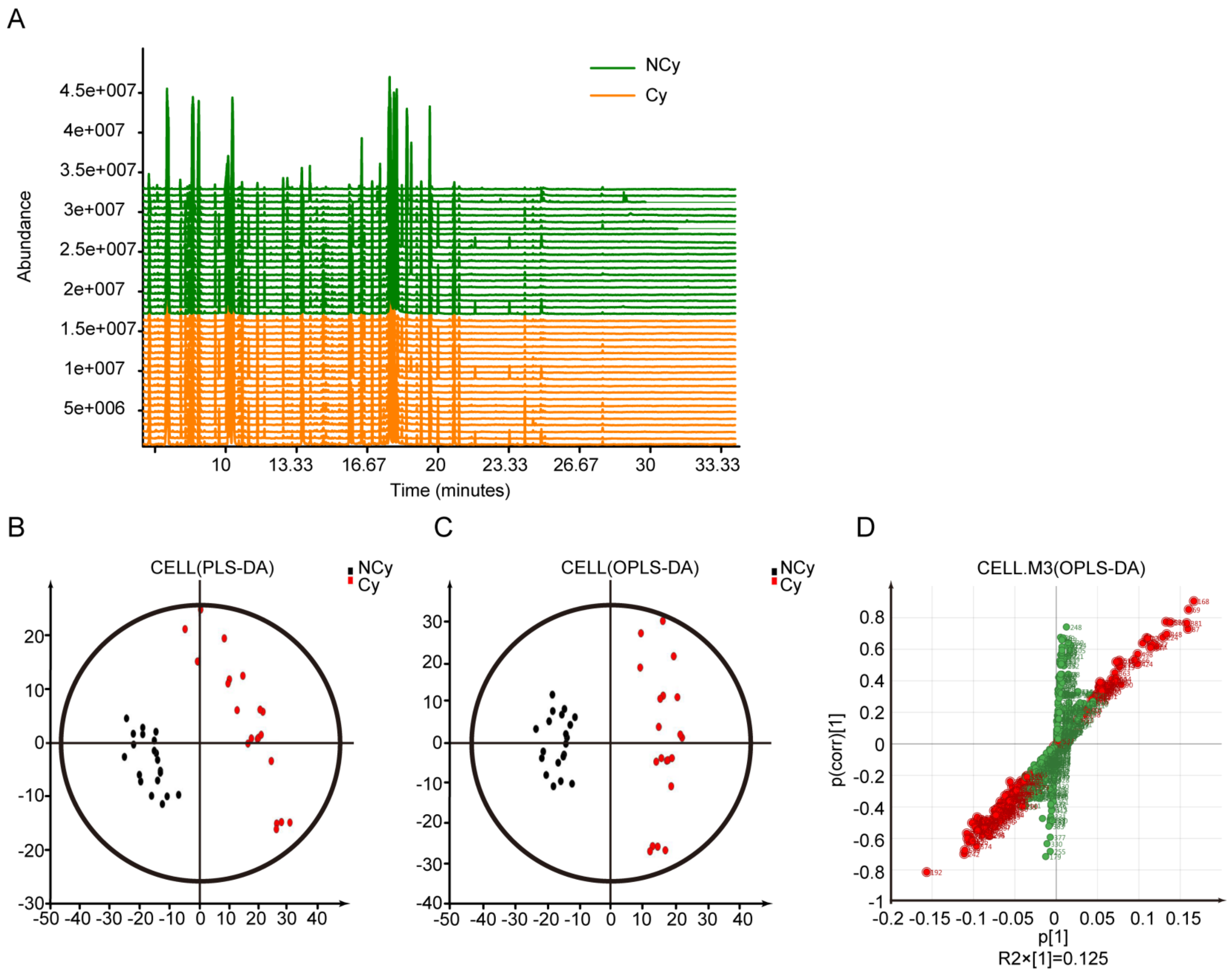

Supplementary Figure 3. Metabolic profiling in BM revealed a significant difference between patients with and without CCHD. (A) GC-TOF/MS total ion current (TIC) chromatograms of bone marrow supernatant from the NCy and Cy groups (n=20 per group) identified 543 valid peaks. **(B)** The PLS-DA score plot, $R^2Y=0.958$, $Q^2=0.807$, represented a clear classification between the NCy and Cy groups**. (C)** The OPLS-DA score plot, $R^2Y=0.958$, $Q^2=0.81$, showed a certain difference between the NCy and Cy groups. **(D)** The S-plot of the OPLS-DA mode in the first component, with metabolites with VIP >1 indicated in red.

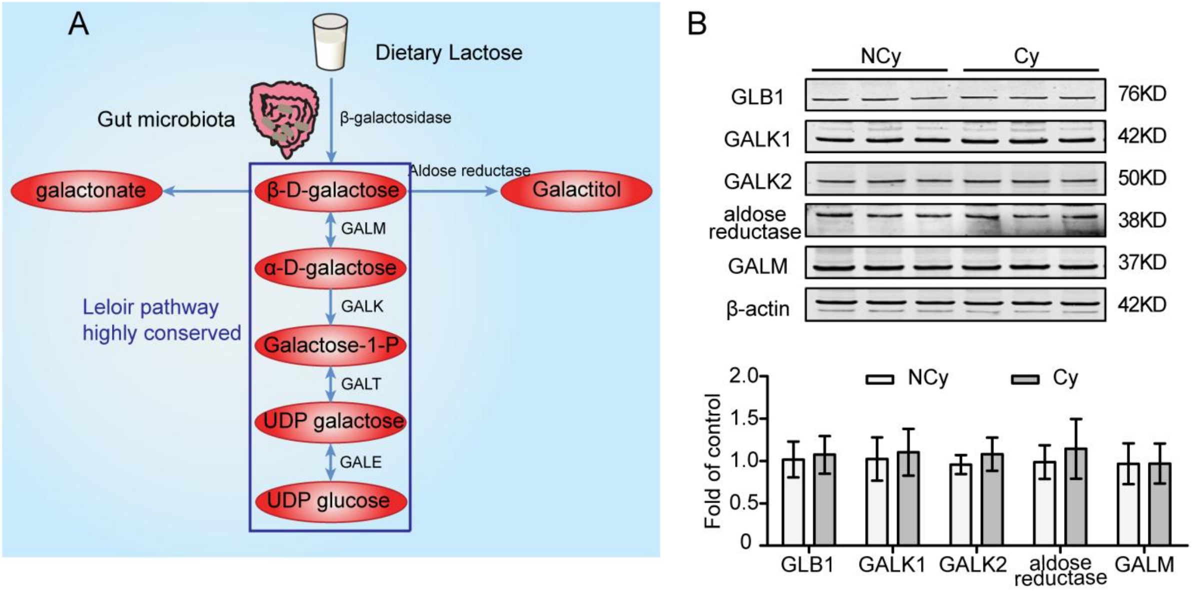

Supplementary Figure 4. The D-galactose metabolism pathway was maintained in human BMSCs remained. (A) The D-galactose metabolism pathway. The Leloir pathway represents the key steps of D-galactose metabolism. **(B)** Western blotting analysis of the protein levels of GALM, GALK1, GALK2, aldose reductase, GLB1 and β-actin in passage-3 human BMSCs. Data are the mean ± SD from 3 independent experiments, and statistical significance was analyzed using the Student's t test.

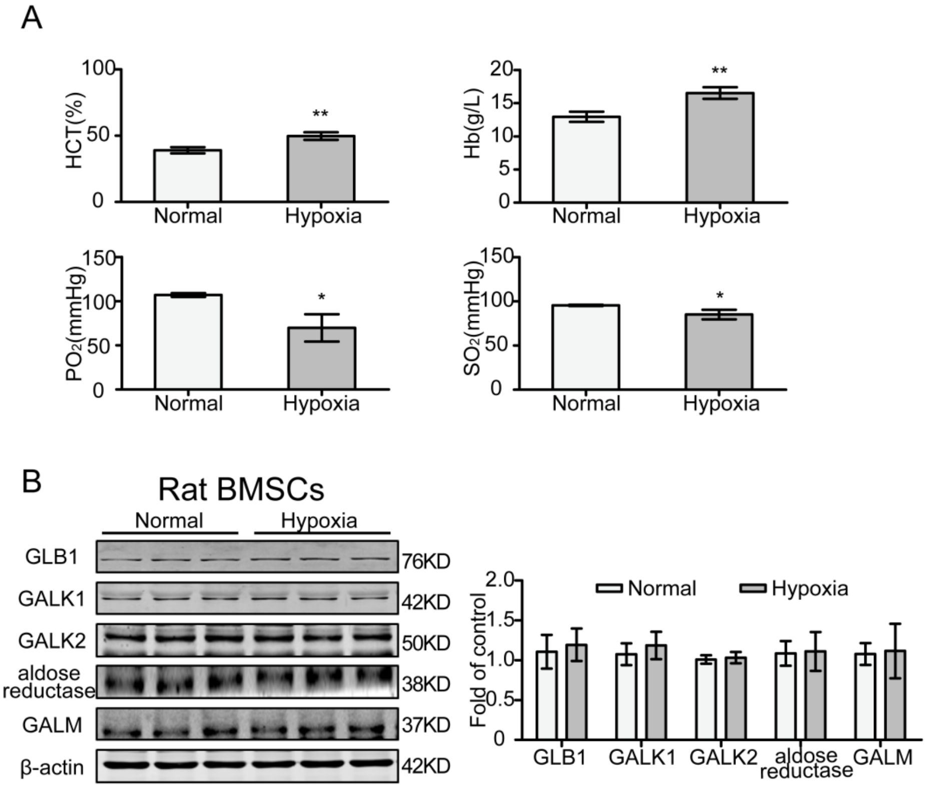

Supplementary Figure 5. Hypoxic rat model. (A) New-born SD rats were housed in a normoxic or hypoxic chamber for 3 weeks, respectively. HCT, Hb, PO_2 and SO_2 were measured. **(B)** Western blotting was used to assess the protein levels of GALM, GALK1, GALK2, aldose reductase, GLB1 and β-actin in rat BMSCs. Data are the mean ± SD from 3 independent experiments, and statistical significance was analyzed using Student's t test (*, $P < 0.05$; **, $P < 0.01$)

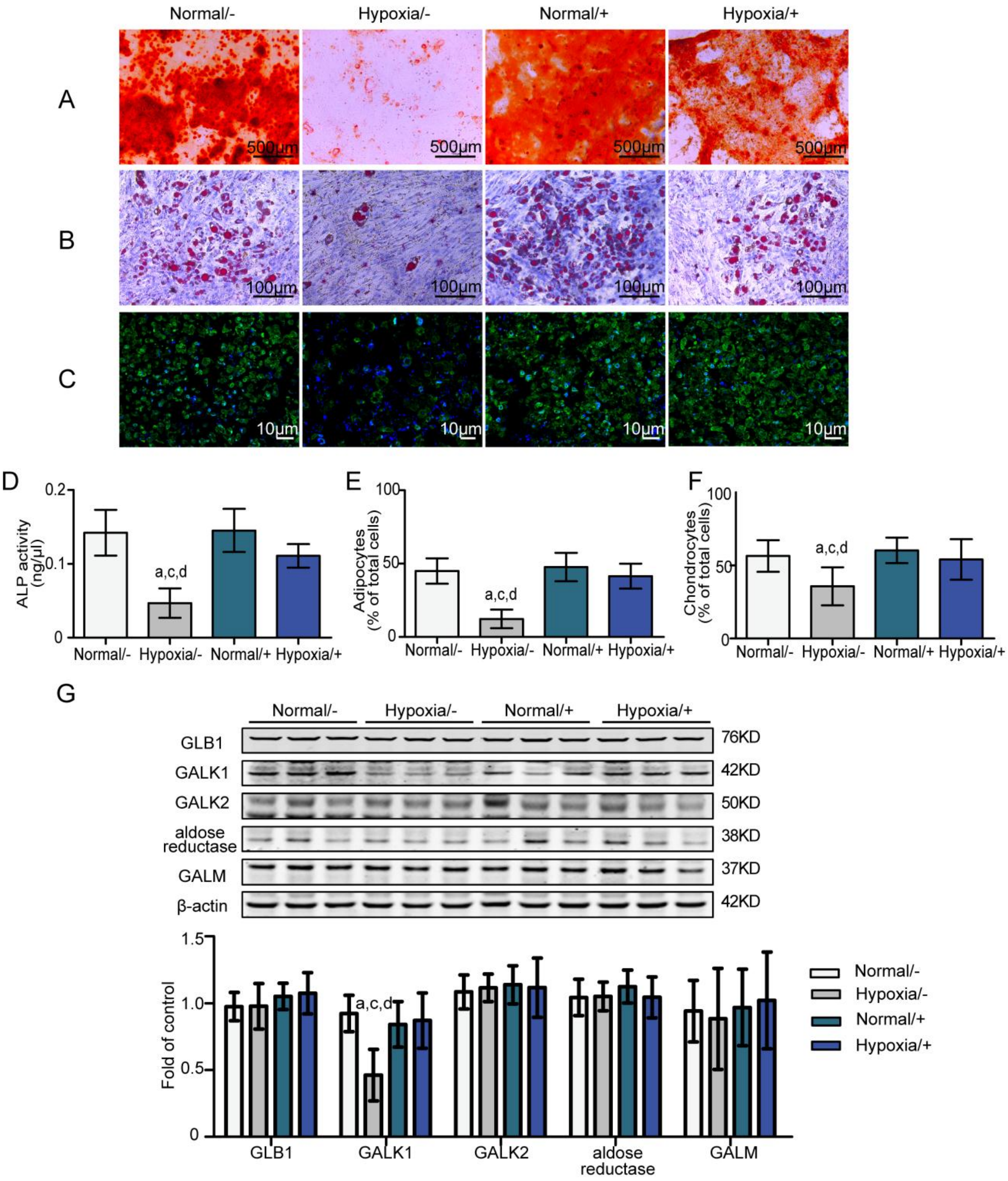

Supplementary Figure 6. *Lactobacillus* supplementation rescued the BMSCs in chronic hypoxic rats from impaired multilineage differentiation potential and reduced liver GALK1 levels. (A, D) Osteogenic differentiation assay of BMSCs from the normal/-, normal/+, hypoxia/- and hypoxia/+ rat groups (n=9 per group). Alkaline phosphatase (ALP) activity assay **(D)** and Alizarin red staining **(A)** were carried out to estimate the osteogenic differentiation potential among the four groups. **(B, E)** Adipogenic differentiation assay for BMSCs. Adipocytes from the four groups were stained with Oil Red O **(B)**, and the adipogenic differentiation ratio was calculated **(E)**. **(C, F)** Chondrogenic differentiation assay for BMSCs. Chondrocytes from the four groups were detected by immunofluorescent staining for type II collagen **(C)**, and the chondrogenic differentiation ratio was calculated **(F)**. **(G)** Rat liver was subjected to western blotting to analyse the protein levels of GALM, GALK1, GALK2, aldose reductase, GLB1 and β-actin. The data shown are the mean ± SD from 3 independent experiments, and statistical significance was analyzed by one-way ANOVA followed by Tukey-Kramer multiple comparisons. a $P<0.05$ compared with Normal/-. b $P<0.05$ compared with Hypoxia/-. c $P<0.05$ compared with Normal/+. d $P<0.05$ compared with Hypoxia/+.

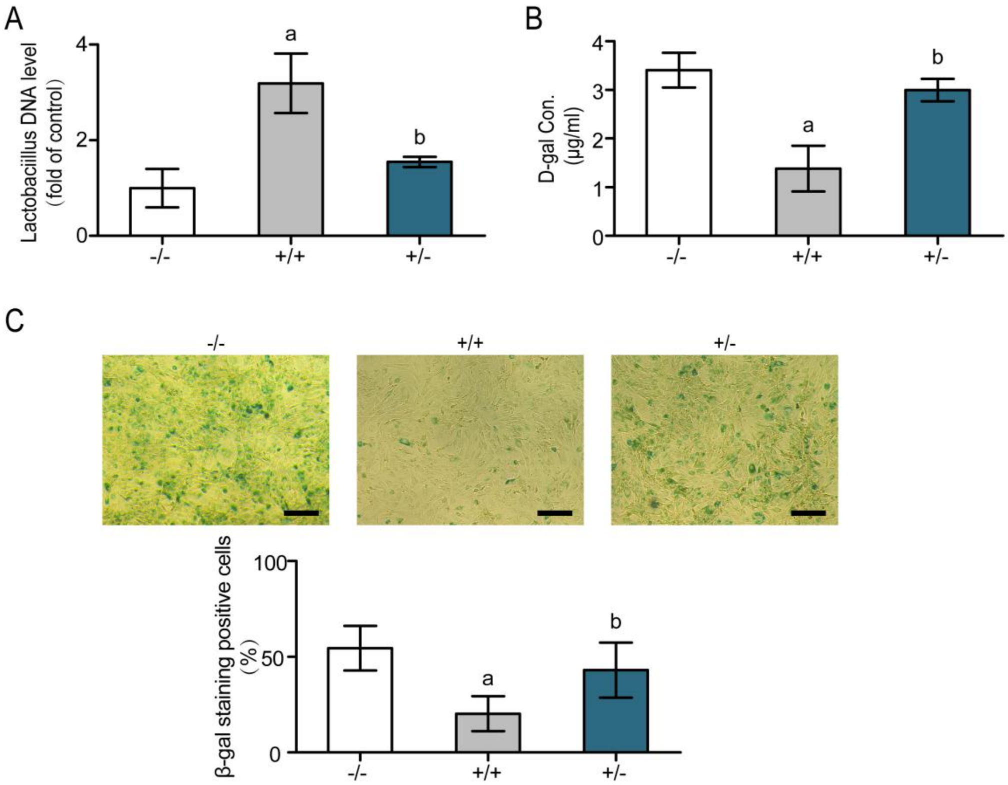

Supplementary Figure 7. Continues supplementary with *Lactobacillus* is necessary for recovery of deficient BMSCs. (A) Rats were divided randomly into three groups (n=6 per group) and housed in the hypoxic chamber for 2 weeks, then supplied with *Lactobacillus* or saline for 3 weeks(-/- group, supplied with saline for entire 3 weeks; +/- group, *Lactobacillus* 1 week followed by saline 2 weeks; +/+ group, supplied with *Lactobacillus* for entire 3 weeks). Stools were subjected to qPCR to assess the *Lactobacillus* content. **(B)** Peripheral blood samples from the rats described in S7A were obtained, and the D-galactose concentrations were measured. **(C)** BMSCs were isolated and cultured to passage 3. SA-β-gal activity was assessed. Representative images are shown (upper). Scale bar, 100μm. The ratio of β-galactosidase-positive cells were calculated. The data shown are the mean ± SD, and statistical significance was analyzed by one-way ANOVA followed by Tukey-Kramer multiple comparisons. a $P < 0.05$ compared with -/- group. b $P < 0.05$ compared with +/+ group.

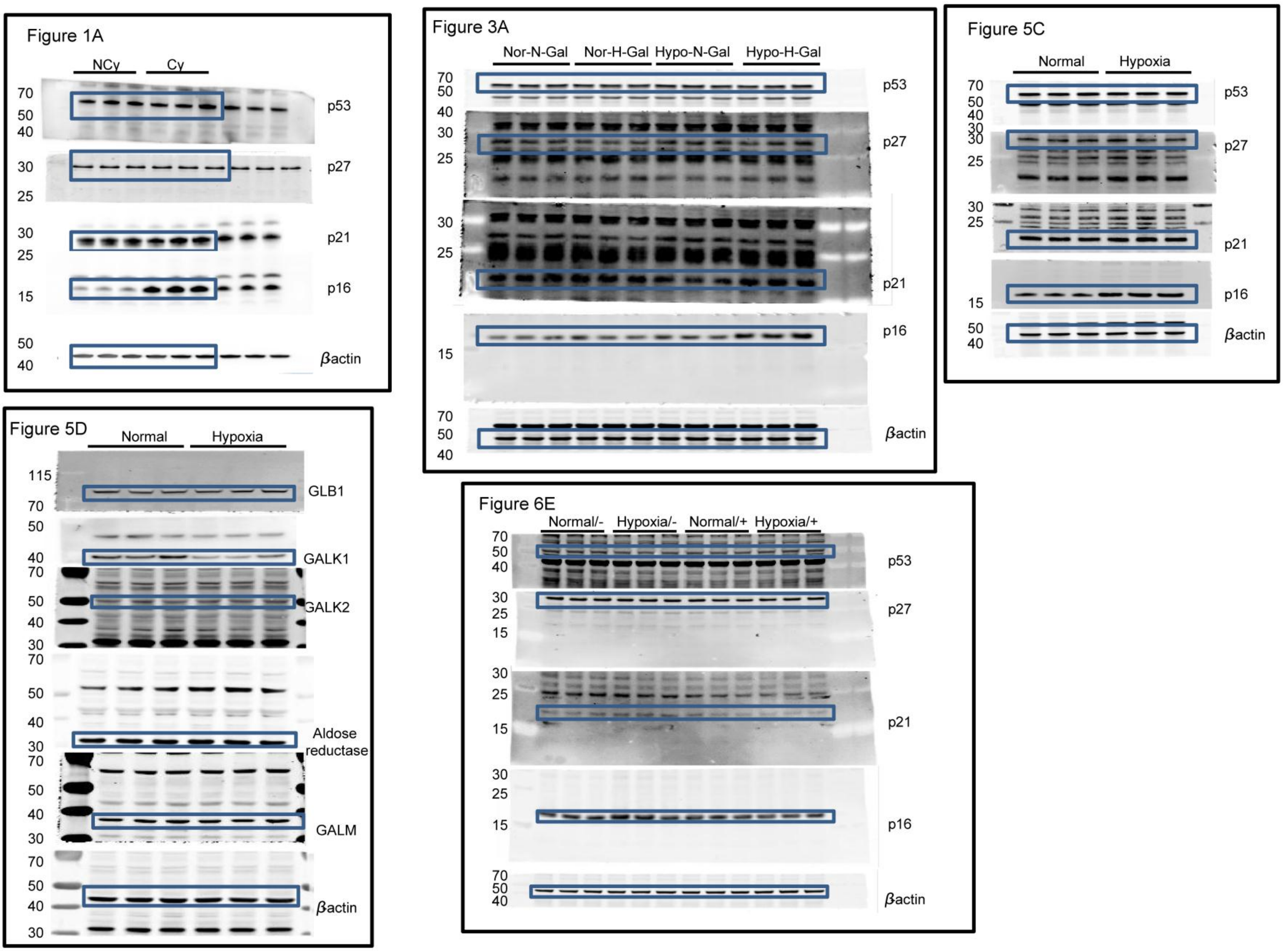

Supplementary Figure S8. Uncropped Western Blots. Uncropped images of all WBs with marker molecular weight indicated, blue boxes indicating cropping used.

Tables

Supplementary Table 1. Identification of Significantly Different Metabolites in Group Cy vs Group NCy

Metabolite	RT	Mass	VIP	P-value	Q- value	FC
D-galactose	17.56	156	2.631	0.014	0.022	47.070
Galactonic acid	18.68	292	1.459	0.023	0.020	30.341
D-erythro-sphingosine	22.53	204	3.056	0.000	0.000	25.255
Creatine	13.95	115	1.286	0.017	0.013	17.463
Taurine	15.37	320	1.536	0.014	0.022	14.399
D-glycerol-1-phosphate	16.10	89	1.234	0.006	0.028	10.422
ribose-5-phosphate	19.76	243	1.570	0.008	0.015	8.265
Orotic acid	16.12	254	1.246	0.021	0.028	8.043
4-hydroxyphenylacetic acid	14.88	355	1.496	0.002	0.006	7.009
Threitol	13.24	144	1.074	0.015	0.023	5.662
Arbutin	23.42	305	1.656	0.026	0.033	5.633
hypoxanthine	16.86	265	2.138	0.031	0.038	5.575
Lactamide	8.83	188	1.239	0.030	0.038	4.736
lactic acid	7.27	146	2.831	0.004	0.010	4.465
fucose	15.81	305	3.674	0.000	0.001	4.375
Alanine	12.30	218	1.397	0.015	0.023	3.123
lactose	24.63	204	1.209	0.047	0.041	2.593
asparagine	15.30	116	1.806	0.045	0.049	2.293
Valine	7.77	174	1.350	0.014	0.022	2.077
3-phosphoglycerate	16.76	211	1.001	0.019	0.027	2.032
Pyruvic acid	7.12	174	1.030	0.043	0.017	1.318
L-glutamic acid	13.69	314	1.070	0.042	0.046	1.271
2-phosphoglycerate	15.89	211	1.787	0.036	0.027	1.168
citric acid	16.90	273	1.038	0.033	0.016	0.862
succinic acid	10.91	129	1.164	0.001	0.008	0.585
malic acid	10.71	147	1.178	0.001	0.003	0.576
Resorcinol	11.82	240	1.563	0.020	0.028	0.557
L-homoserine	15.29	290	1.566	0.005	0.010	0.543
Ornithine	14.58	241	1.716	0.028	0.019	0.476
Erythrose	12.61	117	1.573	0.002	0.006	0.456
trans-4-hydroxy-L-proline	13.61	304	1.360	0.019	0.027	0.454
putrescine	16.08	174	1.420	0.011	0.011	0.451
Galactose-1-phosphate	7.04	229	1.443	0.001	0.018	0.433
methionine sulfoxide	17.47	96	1.509	0.001	0.003	0.407
Salicin	23.31	217	2.223	0.000	0.001	0.405

Metabolite	RT	Mass	VIP	P-value	Q- value	FC
Ethanolamine	10.20	192	2.277	0.013	0.021	0.392
linolenic acid	20.77	243	2.022	0.001	0.003	0.383
Glycine-d5	10.77	145	2.391	0.007	0.014	0.316
gly-pro	18.59	174	1.729	0.001	0.005	0.270
Pyrrole-2-Carboxylic Acid	11.38	240	1.698	0.010	0.018	0.255
D-Arabitol	15.83	306	1.831	0.028	0.036	0.188
uridine	22.59	133	1.528	0.000	0.001	0.176
Catechol	10.93	136	2.433	0.000	0.001	0.166
1,2,4-Benzenetriol	14.50	239	1.111	0.010	0.018	0.159
L-Allothreonine	10.59	144	1.759	0.001	0.003	0.153
serine	11.48	340	2.244	0.005	0.010	0.128
Isoleucine	10.54	231	2.438	0.000	0.000	0.114
Itaconic acid	11.25	147	3.648	0.000	0.000	0.069

RT = retention time. FC = fold change, mean value of peak area obtained from Group Cy /mean value of peak area obtained from Group NCy.
